# REIMAGINING AGING: LESSONS ON HEALTHY AGING FROM AARP

**DOI:** 10.1093/geroni/igac059.904

**Published:** 2022-12-20

**Authors:** Teresa Keenan, Cheryl Lampkin

**Affiliations:** AARP, Washington, District of Columbia, United States; AARP, Washington, District of Columbia, United States

## Abstract

This presentation will include insights on healthy aging based on data obtained from multiple recent AARP surveys fielded among nationally representative samples of midlife and older adults. Samples were weighted to reflect population estimates (i.e., gender, ethnicity, etc.). Studies also included oversamples of Black/African American and Hispanic/Latino respondents when possible, enabling enhanced analyses. This multi-analysis presentation will provide valuable insights on healthy aging in general, the impact the COVID-19 pandemic has had on the older populations’ ability to maintain healthy habits, and how our “new normal” suggests the need to reimagine healthy aging. This presentation will showcase exciting new findings from AARP Research on diet, exercise, resiliency, and mental health, among others. Augmented with qualitative data, this presentation will also allow participants to hear the voices of older adults on these issues. Additionally, new data collected in late fall 2022 will provide a preview of up-to-the-minute insights.

